# Generic Information Can Retrieve Known Biological Associations: Implications for Biomedical Knowledge Discovery

**DOI:** 10.1371/journal.pone.0078665

**Published:** 2013-11-19

**Authors:** Herman H. H. B. M. van Haagen, Peter A. C. 't Hoen, Barend Mons, Erik A. Schultes

**Affiliations:** Department of Human Genetics, Leiden University Medical Center, Leiden, The Netherlands; Max Planck Institute for the Physics of Complex Systems, Germany

## Abstract

**Motivation:**

Weighted semantic networks built from text-mined literature can be used to retrieve known protein-protein or gene-disease associations, and have been shown to anticipate associations years before they are explicitly stated in the literature. Our text-mining system recognizes over 640,000 biomedical concepts: some are specific (i.e., names of genes or proteins) others generic (e.g., ‘Homo sapiens’). Generic concepts may play important roles in automated information retrieval, extraction, and inference but may also result in concept overload and confound retrieval and reasoning with low-relevance or even spurious links. Here, we attempted to optimize the retrieval performance for protein-protein interactions (PPI) by filtering generic concepts (node filtering) or links to generic concepts (edge filtering) from a weighted semantic network. First, we defined metrics based on network properties that quantify the specificity of concepts. Then using these metrics, we systematically filtered generic information from the network while monitoring retrieval performance of known protein-protein interactions. We also systematically filtered specific information from the network (inverse filtering), and assessed the retrieval performance of networks composed of generic information alone.

**Results:**

Filtering generic or specific information induced a two-phase response in retrieval performance: initially the effects of filtering were minimal but beyond a critical threshold network performance suddenly drops. Contrary to expectations, networks composed exclusively of generic information demonstrated retrieval performance comparable to unfiltered networks that also contain specific concepts. Furthermore, an analysis using individual generic concepts demonstrated that they can effectively support the retrieval of known protein-protein interactions. For instance the concept “binding” is indicative for PPI retrieval and the concept “mutation abnormality” is indicative for gene-disease associations.

**Conclusion:**

Generic concepts are important for information retrieval and cannot be removed from semantic networks without negative impact on retrieval performance.

## Introduction

The growth of scientific literature in the biomedical and life sciences has surpassed the capacity of human comprehension. Without some means of automatic data integration, increasing amounts of valuable information will remain lost in plain sight, relevant and novel implications (i.e., novel associations) will go untested, and expensive experimental research projects will be needlessly replicated [1]. In response, numerous text-mining based integration tools have been developed for automated information retrieval, extraction, and inference [2]–[4]. These tools are often developed and benchmarked in retrospective studies, but have potential for knowledge discovery.

We use a text-mining and inference system based on concept profiles to expose novel and relevant associations between concepts from biomedical literature. This information retrieval system has been shown in retrospective studies to rediscover gene-chemical, protein-protein, and gene-disease associations in some cases years before they were explicitly stated in the literature [5], [6]. Concept profiles have also been shown to predict protein-protein interactions that were subsequently validated experimentally [7].

Concept profiles for information retrieval and knowledge discovery are generated in a three-step process. First, a large text corpus (in this case 10 million MEDLINE abstracts) is indexed using a custom thesaurus, mapping and disambiguating terms to specific biomedical concepts. The concepts belong to a curated compilation of existing biomedical ontologies and cover diseases, symptoms, tissues, biological processes and other biometrically relevant semantic types. Second, for each concept, a weighted list (profile) of all other concepts is constructed from the observed co-occurrence frequency in each abstract. For example, gene and disease concepts typically have hundreds of other concepts in their profiles, and some have thousands of concepts. Third, the number and weights of the shared concepts between two concept profiles is used to determine the strength of the association. The concept profile match score can be computed using various vector-matching methods.

Importantly, concept profiles allow the individual contribution of each shared concept to the overall match score to be quantified (Table 1). Expert users can then scan this list of shared concepts as an aid in the subsequent rationalization of the inferred association. In turn, this can help the researcher formulate testable hypotheses [7]. The list of shared concepts is thus a potential resource for knowledge navigation and discovery.

**Table 1:** The 20 highest and 20 lowest ranking shared concepts between the proteins CAPN3 and PARVB with the percent contribution of each concept to the overall match score.

Rank	Overlapping Concept	Contribution (%)
1	DYSF	82
2	CAPN2	4
3	LGMD2B	3.6
4	Limb girdle	3.3
5	Actinin	1.9
6	muscular dystrophy	1.3
7	CAPN1	1.1
8	CAPNS1	0.57
9	CAST	0.35
10	LAMA2	0.31
11	congenital muscular dystrophy	0.23
12	CAV3	0.22
13	Sarcolemma	0.15
14	LGMD1C	0.12
15	Z line	0.12
16	skeletal muscle structure	0.035
17	positional cloning	0.030
18	Skeletal system	0.028
19	Skeletal Myocytes	0.023
20	Cytoskeleton	0.021
262	Wills	7.5E-07
263	Cloning	7.4E-07
264	Activities	7.1E-07
265	Others	6.0E-07
266	Evolution	5.6E-07
267	physical assessment findings	5.5E-07
268	cellular targeting	5.5E-07
269	Adult	5.4E-07
270	Laboratory Procedures	4.7E-07
271	Clone Cells	4.4E-07
272	Restricting	3.8E-07
273	Near	3.5E-07
274	Extracellular	3.5E-07
275	cell differentiation process	3.3E-07
276	Collagen	2.8E-07
277	Pathogenesis	2.3E-07
278	Biologic Development	2.2E-07
279	majority	1.5E-07
280	pathogenesis	1.3E-07
281	Event	8.6563E-08

The contribution is calculated as a percentage of an individual product between 2 concepts divided by the inner product (which is the sum of all individual products). This inferred association from text-mining was subsequently validated as a physical protein-protein interaction *in vitro* [7].

The list of shared concepts has some general features. First, we find in general that the first few top-ranking concepts account for 90% or more of the overall match score. The remaining concepts contribute only a tiny fraction but there are many more of them (i.e., hundreds or thousands). For example, Table 1 shows the top 20 highest and 20 lowest ranking shared concepts between the two protein concepts ‘CAPN3’ and ‘PARVB’, along with their contribution to the overall match score. These two proteins were correctly predicted to be interacting by our system, as confirmed by subsequent experimental studies [7]. Furthermore, although concepts near the top of the list tend to have specific meaning and obvious relevance, concepts near the bottom are often interpreted by expert users as being generic or even ‘useless’ or ‘disturbing’, and are perceived by biomedical experts to add little or no information that aids in rationalizing the putative association.

In order to better understand the roles played by specific and generic concepts and in an effort to avoid concept overload and provide the end users with a shorter and more relevant list of shared concepts, we incrementally filtered generic concepts from the network while measuring the impact on information retrieval. First, following previous research [7] we constructed concept profiles for human proteins, and retrieved protein-protein pairs having high match scores. We benchmarked these associations against known sets of protein-protein interactions. Then we rigorously define ‘generic’ and ‘specific’ based on the statistical weights and connectivity properties of the semantic network of concepts given by the concept profiles. Lastly, we filter the generic information from the network by removing low-weight connections (edge filtering) or concepts having high-degree of connectivity (node filtering). In each case, we find that the removal of generic concepts from the network decreased PPI retrieval performance. As a control, we also perform the inverse filtering i.e., we removed specific concepts and measured the PPI retrieval performance of networks composed of generic concepts alone. Surprisingly, networks built from only generic concepts had comparable performance to the unfiltered networks in PPI retrieval. Apparently generic concepts, or combinations of generic concepts, play a vital role in information retrieval even when they represent to the expert user no obvious relevance.

## Methods

### Text-Mining

The Open Source concept recognition software Peregrine scans free-text and resolves homonyms and maps ambiguous terminology and spellings to unique biomedical concepts [8]–[10] (software available at https://trac.nbic.nl/data-mining/). Peregrine uses an extensive custom thesaurus of 640,016 biomedical concepts based on the Unified Medical Language System [11], augmented with concepts from Entrez-Gene [12], Online Mendelian Inheritance in Man [13], UniProt [14], and the Human Gene Nomenclature Database [15]. Using Peregrine, over 10 million MEDLINE documents (titles, Medical Subject Headings, and abstract text) were indexed from January 1980 to December 2009.

### Building Concept Profiles

A concept profile is an M-dimensional vector w_i_ = (w_i1_,w_i2_,…,w_iM_) where *i* a particular concept, and M is the number of concepts in the thesaurus. A concept must occur in a minimum of 5 abstracts before a concept profile is created [9]. The weight w_ij_ for a concept *j* in this profile indicates the strength of its association to the concept *i*. The weights are computed from pair-wise concept-concept co-occurrence frequencies within individual abstracts. Given concepts X and Y, co-occurrence is characterized by 4 contingencies: they may both occur, neither may occur, or one may be present without the other. An association between X and Y is computed from this 2×2 contingency table using a measure of mutual information called the symmetric uncertainty coefficient, U(X_i_,Y_j_) [9], [16], where ‘H’ is entropy.
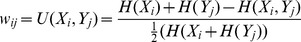
(1)The uncertainty coefficient gives extra weight to those concepts that have specific associations. As an example consider the concept DMD (the gene) and the disease Duchenne Muscular Dystrophy. In the vast majority of MEDLINE abstracts, both concepts will be absent. However, there will still be many abstracts where these concepts co-occur. Relatively few abstracts will mention one concept but not the other. The contingency table reflecting these co-occurrences will result in a high association between DMD and Duchenne as computed by the uncertainty coefficient. In contrast, the concepts ‘human’ and DMD will yield a very different contingency table and association score. In this case, ‘human’ is a generic concept and there will be many abstracts where human and DMD occur together, but also many other abstracts where human occurs without DMD. For contingency tables with generic concepts the uncertainty coefficient will yield a low association score.

### The PPI Weighted Semantic Network

Using Peregrine, the thesaurus and the MEDLINE corpus 11,541 concept profiles for human proteins could be constructed. Each concept found in these profiles was stored together with its weight, creating a weighted semantic network of 158,487 individual concepts. Although the network was constructed for protein concept profiles this network contain concepts of any semantic type.

### Concept Profile Matching

Using concept profiles, we established associations between concept pairs based on the similarity of their concept profiles [7], [9]. Concept profiles can be treated as vectors of weights, where the weights are values derived from the Uncertainty Coefficient. The similarity between two concept profiles A and B can thus be computed by taking the inner product over the weights in the vector. The inner product increases with an increasing number of shared concepts.

### Benchmark Dataset

We use protein-protein interactions (PPI) from the Human Protein Reference Database (HPRD) to serve as a test set of established PPIs. HPRD FLAT_FILES_072010 was downloaded from hprd.org and 37,067 PPIs were extracted. Of these, 32,333 could be mapped to concept profiles. Each match score was normalized to percentile rank scores by comparing each match score to a frequency distribution of match scores constructed from randomly sampled protein pairs.

We made a second test set of gene disease associations from OMIM. We downloaded the morbidmap file from the NCBI website. We selected only diseases and genes having unambiguous OMIM identifiers. In total we obtained 1,800 known gene disease associations. As a reference we constructed a set of 10,000 randomly selected gene-disease pairs from our thesaurus. The diseases are of semantic type ‘syndrome’, or ‘disease’.

### Analysis

We use standard information retrieval measurements to validate the performance of the weighted semantic network [7]. Expectations are that established PPIs will rank higher than novel (but meaningful) protein-protein associations, which in turn are higher ranked than random (meaningless) protein-protein associations. We compute the Area Under the Curve (AuC) of the Receiver Operating Characteristic (ROC) as an indication of the relative ranking of known and unknown associations. A ranked list where first all the established knowledge is shown will have an AuC of 1. A ranked list where the unknown and known information is shown in no particular order (as if it would be random) will have an AuC of 0.5.

### Defining Concept Specificity

**Figure 1:**
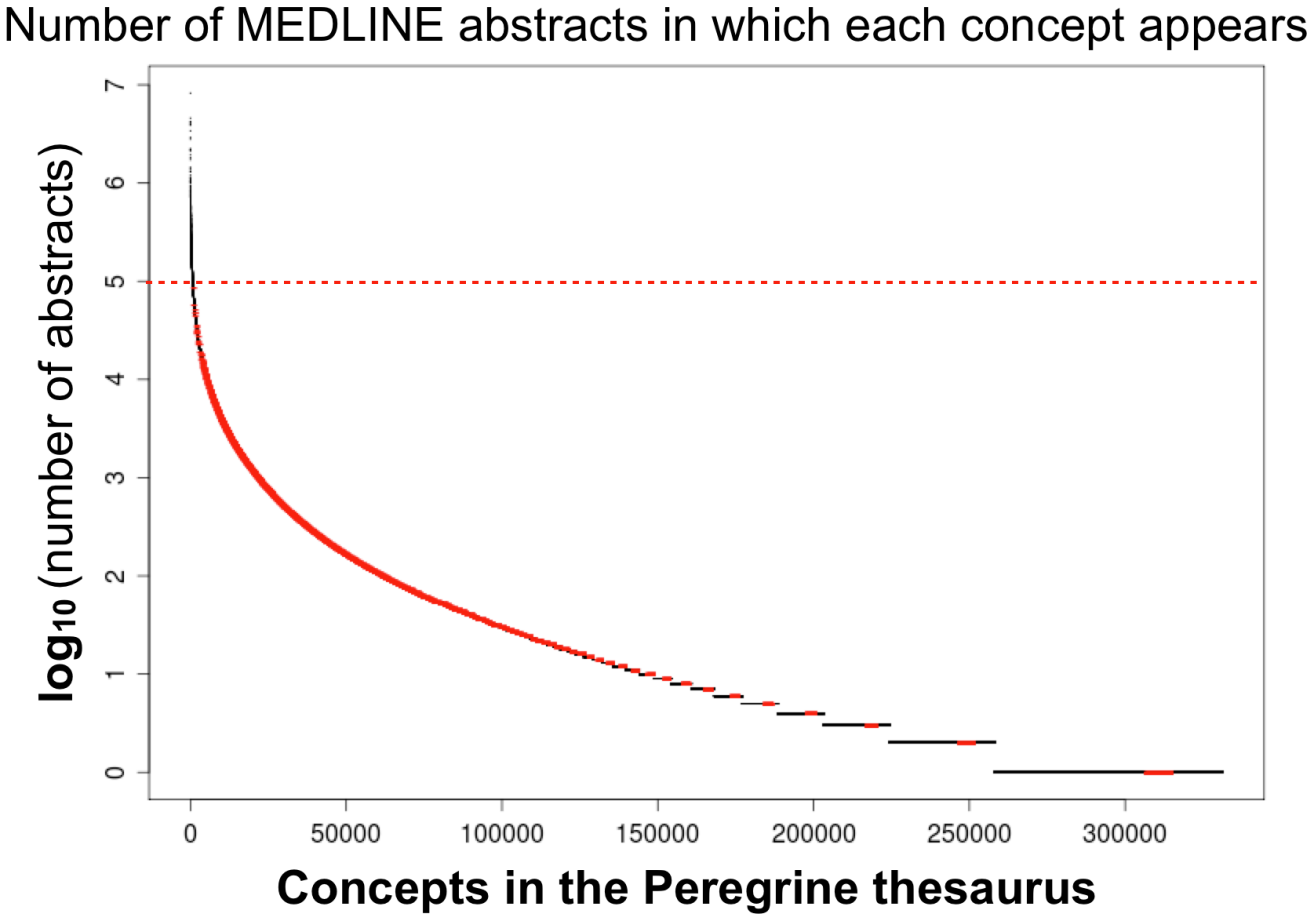
Concepts in the thesaurus ranked by the number of abstracts in which they appear in the MEDLINE text corpus. Generic concepts appear in a large number of abstracts while specific concepts, such as proteins (red points below log 5) tend to occur in a smaller number of abstracts. Not plotted are the 308,656 concepts having no occurrence in MEDLINE abstracts.

Concepts may be specific or generic. Intuitively, we say the concept ‘Homo sapiens” is generic because it is found throughout MEDLINE (appearing in 8,231,081 abstracts) and in association with many different concepts. On the other hand, the protein CAPN3 is specific in that it is found in a smaller number of abstracts (350) and tends to be associated with a narrower range of concepts. To measure the specificity of a concept we consider three attributes:

1. The number of abstracts in which the concept appears: We computed for each concept in the thesaurus the number of abstracts in which it appears. The distribution approximates a power-law (Figure 1). The top of the rank-ordered list is dominated by concepts that appear intuitively to be generic (Table 2). More specific concepts, such as protein names are found lower in the list (below 5 on the log scale). For example, the first instance of a protein (TNF tumor necrosis factor) occurs at rank 871, and it occurs in 85,002 abstracts. (Figure 1). Fifty two percent of the concepts in the list do not appear in any MEDLINE abstract (these are largely complex chemical names and non-human proteins).
2. The number of other concepts to which the concept is connected (network degree): It is reasonable to consider that generic concepts will have high degrees in the network. Since the PPI weighted semantic network consists of 11,541 protein profiles the maximum degree, if that protein appears in each protein profile, will be 11,541.
3. The weights between any two concepts in the network. As discussed above the uncertainty coefficient computes weak associations between two concepts when at least one of two concepts is generic. For example, the association that establishes DMD as a human gene has low weight because ‘gene’ is generic.

**Table 2:** The 30 highest ranking concepts in the thesaurus by the number of abstracts in which they appear in the MEDLINE text corpus (1980–2009).

Rank	Concept	Number of abstracts
1	Homo sapiens	8231081
2	equus asinus asinus	4578399
3	Male gender	4227525
4	Female	4121931
5	Clinical Trials	4108241
6	Scientific Study	3906753
7	DICOM Study	3906024
8	Animals	3404900
9	Patients	2907515
10	Adult	2797586
11	Therapeutic procedure	2225147
12	Aging	2184314
13	Age	2184279
14	Middle Aged	2119368
15	Analysis	1929201
16	Others	1815242
17	Cells	1729989
18	Time	1432880
19	Reported By	1419865
20	Lowing (vocalization)	1371831
21	Indicated	1313043
22	Does not	1302387
23	Activities	1201045
24	Measures	1138383
25	etiology	1134433
26	Methods	1110329
27	Laboratory Procedures	1080545
28	Evaluation procedure	1062532
29	Diagnosis	1052395
30	Related	1048459

These top-ranking concepts appear to be generic.

### Filtering nodes or edges from the network

We filter generic information from the network by either removing generic concepts (node filtering) or by removing an association from the network based on the association strength (edge filtering). In node filtering, we used different filter cut offs ranging from 0 to a maximum of 11,541, with step sizes of 500. In edge filtering, we set thresholds in increments of 0.5 on the log scale. As a more intuitive control, we also filter concepts from the network if that concept appears in a number of abstracts equal to the cut-off. The threshold for number of abstracts is incremented in steps of 0.5 on the log10 scale, over the range from log 0 to 7. These filtering techniques remove generic information from the network, creating a smaller network enriched in specific concepts. However, we also performed the inverse filtering, i.e., removing specific information from the network, thus creating a network comprised of generic information. In all cases, we evaluate the ability of the networks to retrieve PPI using the AuC value of the appropriate ROC.

## Results

### Filtering

**Figure 2:**
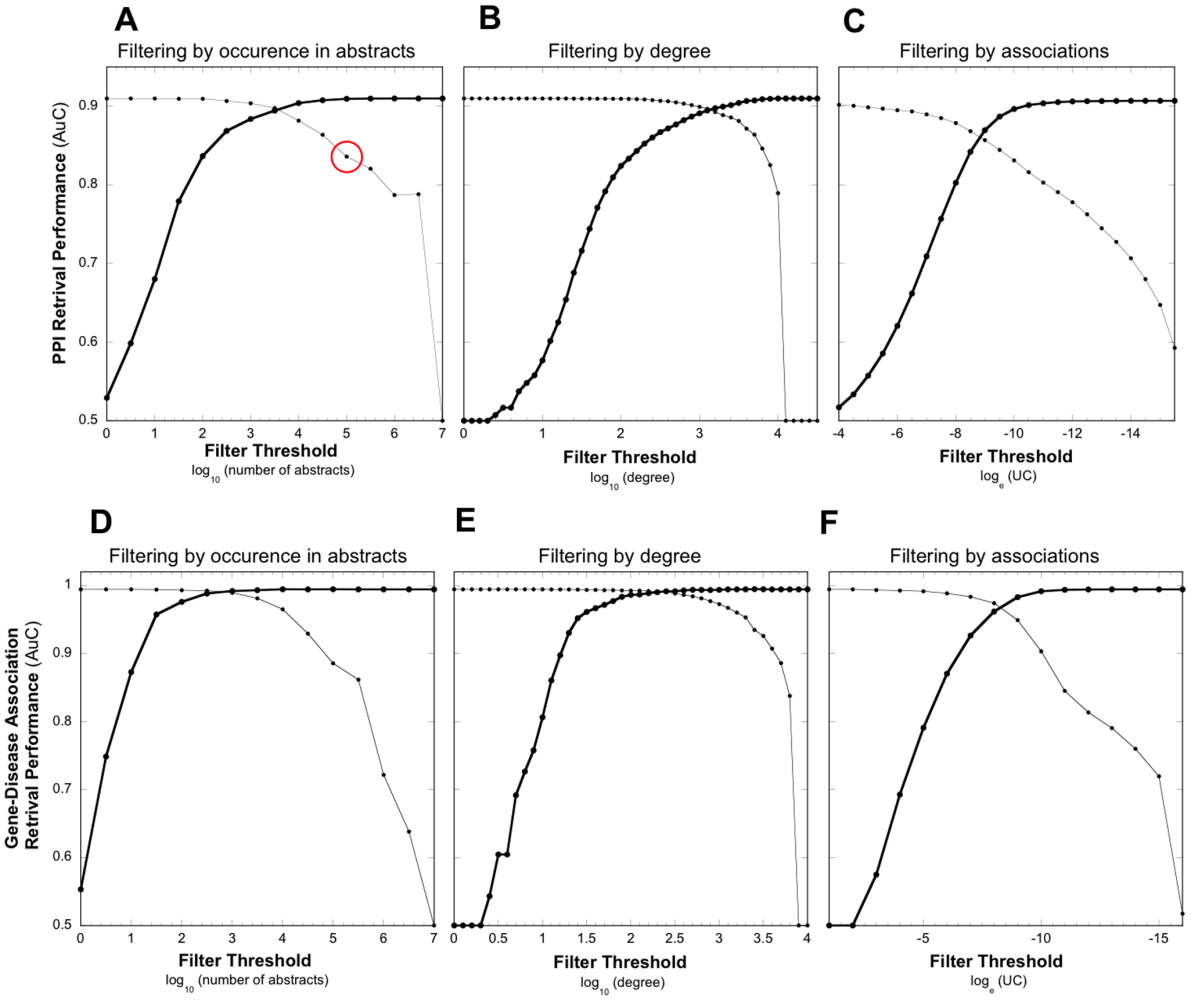
The impact of three different filtering methods on the retrieval performance of the weighted semantic network. A–C, PPI retrieval performance (true positive rate or recall) is measured as the Area under the ROC Curve (ordinate). Panels D–F retrieval performance for known gene-disease associations. An AuC value of 0.5 indicates no retrieval power above random expectations. The weighted semantic network is filtered by incrementally removing generic information (heavy curve) from right to left or by incrementally removing specific information (inverse filtering, light curve) from left to right. Filter Threshold is indicated on the abscissa. Panels A, B, D, and E represent node filtering approaches while panel C and F represent edge filtering (see Method section for details). The red circle in panel A indicates the PPI retrieval performance (0.83) for a network where 99.52% of the nodes have been removed (i.e., all concepts occurring in 100,000 abstracts or fewer).

Figure 2 shows how the PPI retrieval power of the semantic network was impacted when filtering generic information. In each case, the unfiltered network is depicted in the upper right corner of the plot (having an AuC value just above 0.9). As an increasingly more stringent filter threshold was applied from right to left, the performance of the networks (depicted by the heavy curve) at first experienced relatively little change. However, in each case a threshold was eventually reached that initiated a precipitous drop in performance toward the AuC value of 0.5 (i.e., where a network would have no discovery power above random expectations). For example, in Figure 2A, a threshold value of 5 (on the log scale) means concepts that occur in more than 100,000 abstracts were removed from the network.

Although the quantitative features of the curves are distinct, they nonetheless share the similar two-phase behavior of initial robustness to filtering, followed by a dramatic loss of performance. That generic concepts or associations can to some extent be removed from the semantic network with only minimal loss of performance is usually interpreted as an indication that generic concepts carry little or no information required for PPI retrieval, and it is the specific concepts or associations that are most valuable for concept discrimination, retrieval and inference. The dramatic loss of performance occurs only when thresholds are so severe that specific information is itself removed from the network.

To directly test this hypothetical interpretation, we also evaluated the PPI retrieval performance of the inverse filtering process. Rather than removing generic information (moving the threshold from right to left), we removed specific concepts and associations (moving the threshold from left to right). Thus, the points in the far left upper corner of the plots represent the unfiltered original network while points to the right (following the light curves) represent semantic networks increasingly enriched in generic information. For example, in Figure 2A, a threshold value of 5 (in this case) means specific concepts occurring in less than 100,000 abstracts were removed, creating a network enriched in generic concepts found among a very large number of abstracts. The expectation was that without the discriminating power of specific information, PPI retrieval performance should be nil.

To the contrary, we found that generic networks retained substantial PPI retrieval performance (light curves Figure 2). Although not as pronounced, the inverse filter curves also display two-phase behavior of robustness then collapse. Catastrophic failure of the network to retrieve PPIs occurs only at extreme filter thresholds at the far right-hand side of the plots (on the log scale, 6.5 for abstracts, 3 for node degree, and −11.5 for associations).

Comparing the filtering and inverse filtering performance curves on the same plot reveals the counterintuitive, but valuable contribution of generic information in PPI retrieval. For example in Figure 2C, when filtering generic links (heavy curve) the network performance drops to 0.6 at the association threshold of −6 on the log scale. The network, though enriched in specific links, has apparently become too sparse for information to be effectively integrated for PPI retrieval. Yet, the specific links that have been removed at this threshold (light curve) demonstrate an AuC value close to the original network. Similar behavior is found when filtering on the basis of occurrence in abstracts and node degree. Clearly, generic information is capable of the retrieval of PPIs.

To validate this curious finding, we repeated this analysis using a benchmark dataset gene-disease associations (See method section for details). The impact of the three filtering methods on the gene-disease retrieval performance is depicted in Figure 2D–F, and the curves have both qualitative and quantitative similarity to those for PPI retrieval performance. This confirms that our initial results for PPIs were not a special case limited to that semantic type or is an artifact of the text-mining system. Apparently, a relatively small set of the most generic concepts has the capacity to discriminate both PPIs and gene-disease associations. As there is no a priori connection between the most generic concepts in the semantic network and the particular associations we chose to investigate here, it is likely that generic concepts will exhibit retrieval power for any concept-concept association. That is, the reasonable retrieval performance of generic concepts is a generic property.

### Retrieval Power of the Core Generic Network

To better understand the PPI retrieval power of generic information, we investigated the set of generic concepts that remained after setting a stringent filter threshold. Considering the thesaurus rank-ordered by generic concepts (Figure 1), we observe there are 735 top-ranking generic concepts in the thesaurus above the cut-off of 5 on the log scale. From Figure 2A, we see that a semantic network composed only of these 735 top-ranking generic concepts (indicated by the red circle) nonetheless has an AuC of 0.83. In other words, after removing 153,752 (154,487–735) concepts from the semantic network, which is more than 99.5% of the total, the system continues to demonstrate remarkable retrieval power. As indicated before, the concepts appear intuitively to be generic (Table 2). Clearly, the discriminating power of these generic concepts is held in their tremendous number of links. Even so, at this filter threshold nearly 81% of the edges have been removed.

Although the cut-off of 100,000 abstracts is somewhat arbitrary, the resulting 735 concepts apparently form a core network of generic concepts, of which at least some are effective in PPI and gene-disease retrieval.

**Figure 3:**
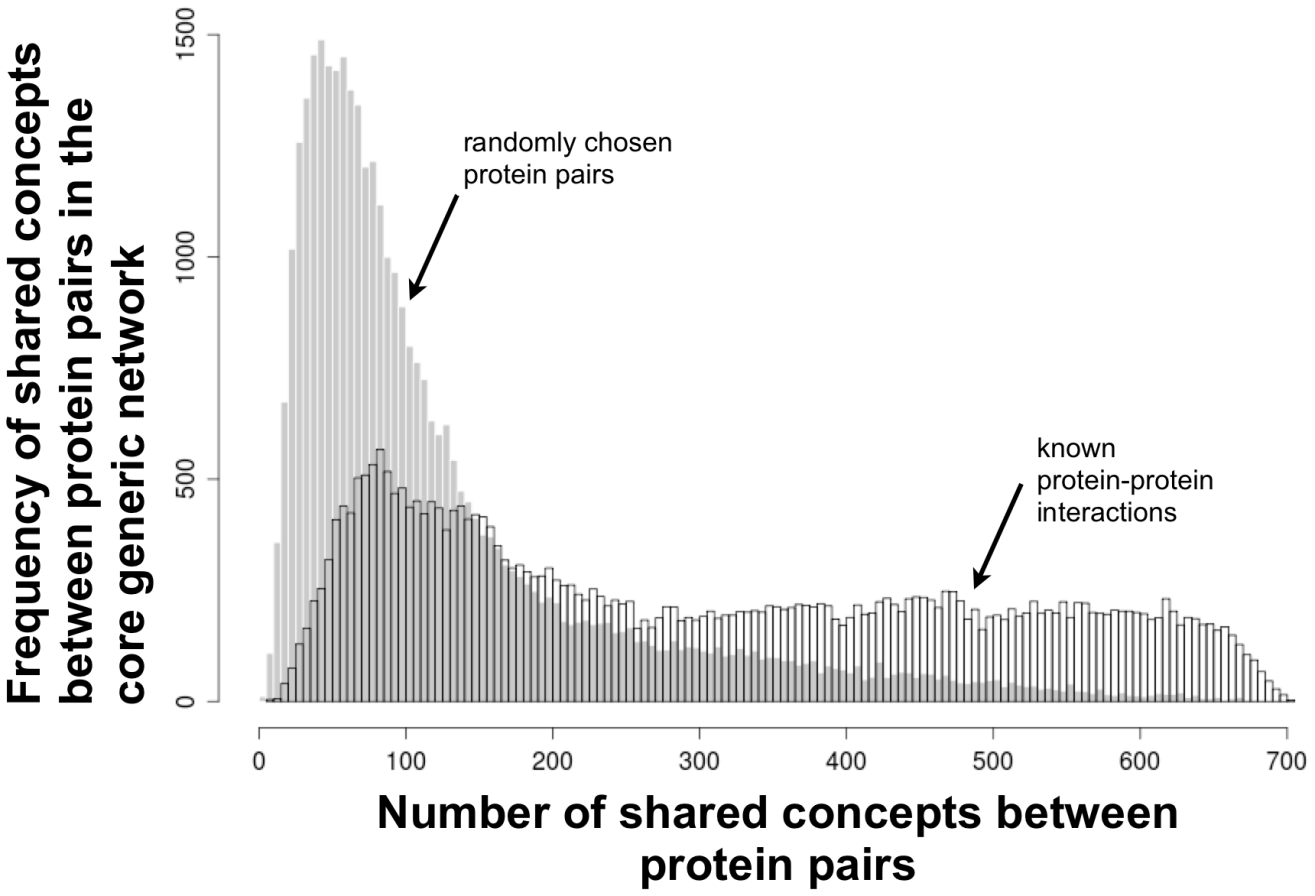
The frequency distribution of core generic concepts shared between PPIs (open bars) is more uniform than is the distribution for randomly chosen protein pairs (grey solid bars). Since the core generic network exists of 735 concepts the number of shared concepts between two profiles can be maximum 735.

To visualize the role of the core network of generic concepts in discriminating PPIs, we determined for each concept the frequency of PPIs that have that concept among their list of shared concepts (the list of shared concepts having an upper bound of 735). For example, the top-ranking PPI (HTT and CASP3) has 631shared concepts from the core generic network. Figure 3 depicts this frequency distribution for the PPIs and an equal number of randomly chosen protein pairs, revealing that these distributions to be distinct: PPIs have a more uniform distribution of generic concepts among their shared concepts than do the random protein pairs. This indicates that concept profiles for proteins having PPIs are enriched in these 735 top-ranking generic concepts.

### Retrieval Power of the Individual Generic Concepts

**Figure 4:**
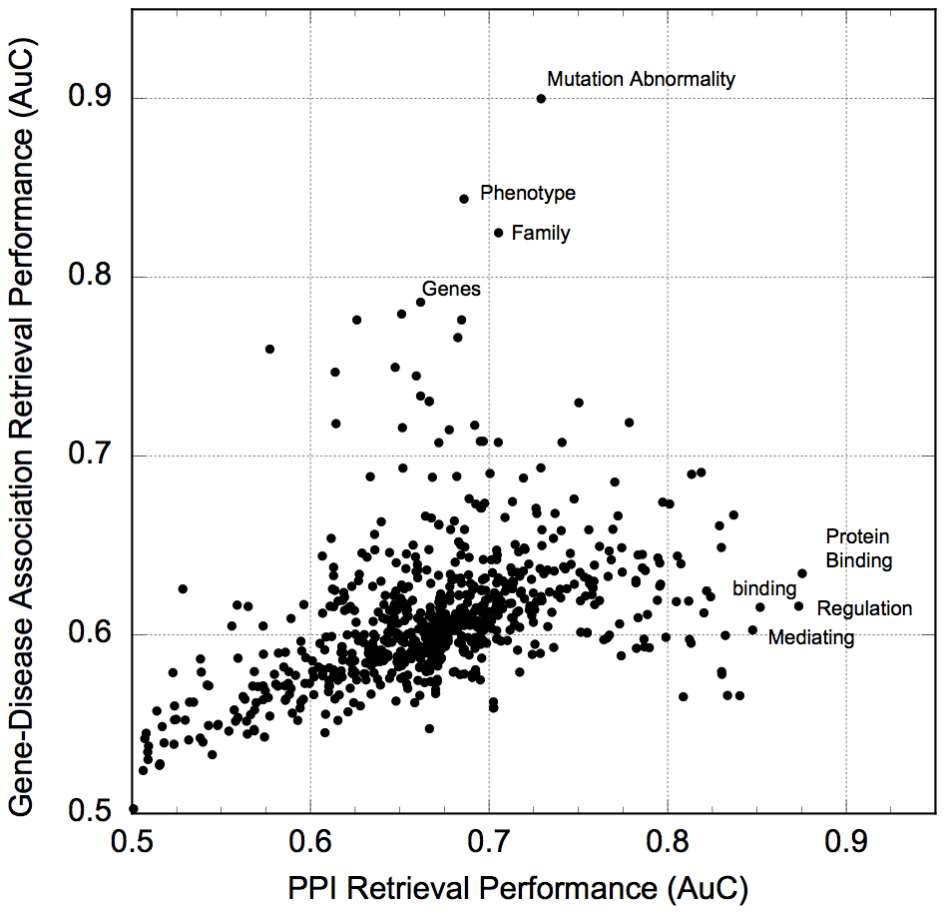
The retrieval power of individual generic concepts. Plotted are the AuC values for 735 individual concepts when retrieving PPI (x-axis) and gene-disease associations (y-axis).

The Core Network of 735 Generic Concepts was identified by inspection of Figures 1 and 2A (red circle). However, in all the plots of Figure 2, comparable retrieval power can be obtained from even more stringent filter thresholds (i.e., even smaller sets of generic concepts). For example, in Figure 2A, AuC values of nearly 0.8 can be obtained from a filter threshold of 6.5 (concepts appearing in 3 million abstracts or more). In this case, there are only 8 generic concepts composing the network, yet they are highly effective in the identification of the PPI benchmark. This result indicates that individual concepts can make a significant contribution to retrieval, and so we pushed this observation to the limit of single concepts. We tested each of the 735 concepts in the Core Generic Network for retrieval of both PPI and gene-disease associations (Figure 4). From this we see that the majority of the concepts do in fact have moderate retrieval performance (AuC around 0.6–0.7). The lowest scoring concept (AuC = 0.5, no retrieval power) is “plants”. On the other hand, a relatively small number of concepts exhibit remarkable retrieval power (AuC 0.8 or above). The top-ranking concepts for PPI and gene-disease retrieval are listed in the plot, and although generic, appear to have special relevance to the retrieval task. For example, the highest-scoring concepts for PPI retrieval are “protein binding”, “regulation”, “binding”, and “mediation”. However, these concepts retrieve gene-disease associations with only moderate performance (AuC values just above 0.6). A similar, but inverse pattern holds for concepts that score highest in gene-disease retrieval. “Mutation Abnormality” which is the 183rd most generic concept, but has obvious relevance to genetic diseases (Auc 0.90) but PPIs less so (AuC 0.73).

**Figure 5:**
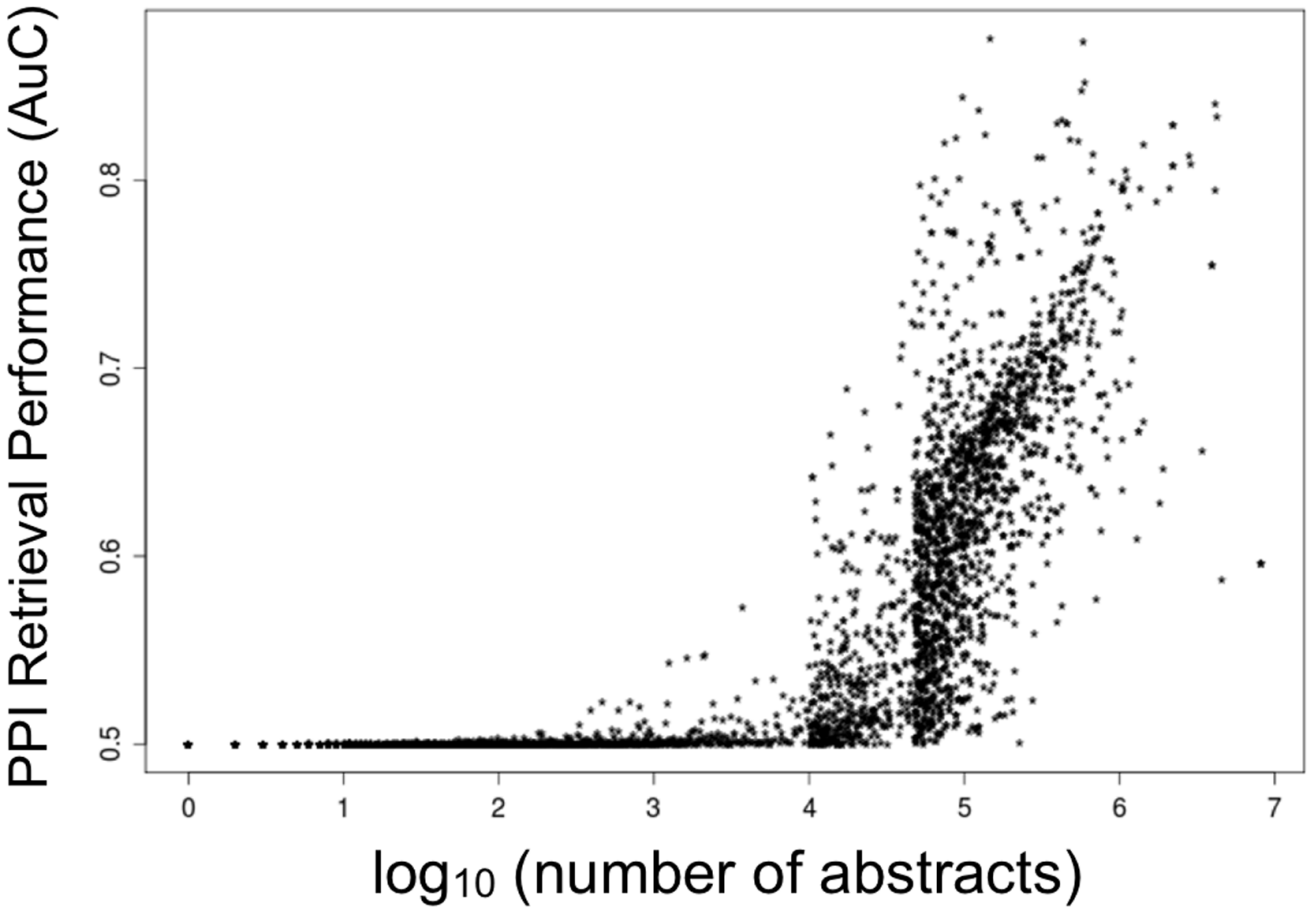
The PPI retrieval power of individual concepts (ordinate) spanning the specific-generic spectrum (log number of abstracts, abscissa). The distribution of number of abstracts in which concepts occur follows a power-law (there are many concepts appearing in only a few abstracts, and few concepts appearing in many abstracts). Hence, the range of abstracts was sampled in three sections (<4, <4.7 <, and the rest) in order to establish clear trends for each case. Above 4.7 all 1431 concepts were evaluated.

To further demonstrate the retrieval power of individual concepts, we expanded the analysis to include concepts from the PPI network spanning the entire range from specific to generic (Figure 5). These results demonstrated a clear trend that more generic concepts indeed have higher retrieval power.

## Discussion

The ultimate aim of network filtering is to optimize inference and guide expert users when navigating the landscape of novel associations. When using concept profile matching to identify strongly associated pairs of concepts, the list of shared concepts creating the association contains both generic and specific information, and this has been used by experts in rationalizing the semantic basis for the associations. To help the user gain more from the list of shared concepts, we felt it was necessary to create lists that had fewer generic concepts and/or prioritized specific and relevant concepts to higher ranks. We also hypothesized that eliminating some generic concepts could lead to improvements in semantic reasoning (as measured by benchmarked true positive rates).

However, the results presented herein indicate that node or edge information, either generic or specific, cannot be filtered from the weighted semantic network without a loss of PPI retrieval performance. This suggests that nearly all the concepts and links in the network are making perhaps small, but still important contributions to the retrieval process. Hence, it is not possible to give users a shorter list without a loss of retrieval and inference power.

Although this result came a surprise, analogous findings appear to have been made other text-mining analyses. For example, it was demonstrated that ‘common words’ such as ‘in’, ‘of’, ‘and’, ‘if’, ‘or’, ‘many’, which can also be regarded as generic, form a ‘backbone structure’ to literary texts and at the same time provide a signature of those texts [17], [18].

In any case, the indispensable role of generic concepts creates a dilemma where, on one hand, we cannot afford to remove more generic elements (nodes or edges) from the network, while on the other hand most of the generic elements will not be meaningful to the human expert. Indeed, in our experience working with biomedical researchers we find that generic concepts are often disturbing to the rationalization process. The question then is how to optimize retrieval and reasoning and at the same time present optimal output for interpretation and rationalization by experts.

We propose to separate the information that is ‘presented to humans’ from the information processed by computer in *in silico* reasoning. We propose to present information to users in ways that are customized to their own expertise. The fact that many more (up to thousands) of concepts have contributed small but essential fractions to the reasoning process should be ‘known to them but not shown to them’. Instead, lists of shared concepts or associations could be prioritized based on concept profiles constructed specifically for the user's expertise (based on, for example, text-mining their own corpus of publications and project proposals). The user could ‘filter’ their personal concept profile and remove the concepts, whether specific or generic, having little relevance to them. This personal (and personalized) concept profile could then be the ‘filter’ when inspecting the output.

This scenario does not only help solve the dilemma of generic concepts for an individual researcher, but it also reflects the use of “social machines” [19] in harnessing diverse expertise. For complex problems that may require multiple experts, personalized concept profiles permit users with different expertise to view the same outputs from unique and potentially complementary points of view.

The results presented herein indicate that node or edge information, either generic or specific, cannot be filtered from the weighted semantic network without a loss of PPI retrieval performance. Although the specificity metrics defined in Section 2.7 are intuitively reasonable, more sophisticated metrics can also be introduced. For example, in addition to degree and weights, we may also consider the heterogeneity in the distribution of weights to any given concept. Presumably generic concepts will have a more uniform distribution of (low) weight edges while specific concepts will have a relatively small number of high weight edges even if they have high degree. We also see that the polar characterization of concepts as either generic or specific is likely too simplistic. For example, the concept glutamate is clearly generic, and occurs in position 632 in the concept profile for the disease concept ‘migraine’. However, migraine researchers have come to see a special role for glutamate in the etiology of the diseases and have an expectation to see the concept ranking high in the concept profile. In this case, the generic concept ‘glutamate’ has a conditional specificity. Without context, glutamate is generic, but in the context of a particular disease (e.g., in association with concepts such as ‘migraine’, ‘aura’ or ‘calcium channel’), glutamate has a new level of relevance and specificity. This conditional specificity may be computed by considering the joint degree or edge weights of glutamate along with its associated concepts. Conditional specificity might be effectively modeled using the cluster coefficient [20]. By using the cluster coefficient, we can begin to model associations not only between concepts, but also between naturally occurring clusters of concepts. In this way, glutamate might have highly ranked associations with migraine, even though it is generic outside that context.

## Conclusion

Generic concepts are characterized by a broad spectrum and a high number of weak associations with other concepts. Herein we investigate the effects of filtering generic concepts on retrieval of PPI. The results indicate that node or edge information, either generic or specific, cannot be filtered from the weighted semantic network without a loss of PPI retrieval performance. This implies that all the concepts and links in the network are making important contributions to information retrieval.
